# Synthesis in the glycosciences

**DOI:** 10.3762/bjoc.6.16

**Published:** 2010-02-22

**Authors:** Thisbe K Lindhorst

**Affiliations:** 1 Otto Diels Institute of Organic Chemistry, Christiana Albertina University of Kiel, Otto-Hahn-Platz 3/4, 24098 Kiel, Germany

All cells are coated in carbohydrates and glycoconjugates. Today, after decades where sugars were regarded mainly as a means of energy storage or simply as molecular material, it is now known that carbohydrates are deeply involved in cellular communication. This awareness of the biological importance of carbohydrates has led to glycosciences becoming an intriguing and fascinating field of interdisciplinary research. However, the structural diversity found in the carbohydrate regime is unparalleled [1] which makes the biological study of carbohydrate recognition and understanding the processes involved rather complicated. In addition, the multivalent nature of most carbohydrate ligands constitutes a special challenge in glycoscience.

Since the isolation of those complex glycans which are active in cellular communication is problematic, oligosaccharide synthesis is an important area of research. Moreover, what Professor Hans Paulsen, one of the greatest exponents of glycoside synthesis, observed in 1982 [2] still holds true today: “*Although we have now learned to synthesize oligosaccharides, it should be emphasized that each oligosaccharide synthesis remains an independent problem, whose resolution requires considerable systematic research and a good deal of know-how. There are no universal reaction conditions for oligosaccharide syntheses”*.

It is therefore not surprising that the majority of contributions collected in this Thematic Series deal with methods, both chemical and enzymatic, for oligosaccharide synthesis.

One approach to deal with the problem of glycoside synthesis is the preparation of so-called glycomimetics. This is a strategy where the glycosidic linkage is substituted by another type of ligation, or where glycosidations are limited to rather simple reactions whilst complexity and multivalency of a complicated target structure are introduced by alternative methods. Examples of such a versatile approach are also presented in this series. Furthermore, carbohydrate chemistry is presented in the context of chemical biology together with organic chemistry making use of known chemical reactions as well as the stereochemical advantages of saccharides to construct novel molecules with unique properties.

It has been a joy to direct this stimulating collection of research from the many areas of the exciting field of the glycosciences. I would like to thank all authors for their excellent contributions. Enjoy reading them!

Thisbe Lindhorst

Kiel, February 2010

## References

[1] Werz D B, Ranzinger R, Herget S, Adibekian A, von der Lieth C-W, Seeberger P H (2007). ACS Chem Biol.

[2] Paulsen H (1982). Angew Chem, Int Ed Engl.

